# Three Decades Single Center Experience of Airway Complications After Lung Transplantation

**DOI:** 10.3389/ti.2023.11519

**Published:** 2023-10-16

**Authors:** R. van Pel, C. T. Gan, W. van der Bij, E. A. M. Verschuuren, J. P. A. van Gemert, C. Van De Wauwer, M. E. Erasmus, D. J. Slebos

**Affiliations:** ^1^ Department of Respiratory Medicine, University Medical Center Groningen, University of Groningen, Groningen, Netherlands; ^2^ Department of Respiratory Medicine, Erasmus University Medical Center Rotterdam, Rotterdam, Netherlands; ^3^ Erasmus MC Transplant Institute, Erasmus University Medical Center Rotterdam, Rotterdam, Netherlands; ^4^ Department of Cardio-Thoracic Surgery, University Medical Center Groningen, University of Groningen, Groningen, Netherlands

**Keywords:** lung transplant, anastomosis, bronchoscopy, airway complications, airway stent

## Abstract

Post lung transplantation airway complications like necrosis, stenosis, malacia and dehiscence cause significant morbidity, and are most likely caused by post-operative hypo perfusion of the anastomosis. Treatment can be challenging, and airway stent placement can be necessary in severe cases. Risk factors for development of airway complications vary between studies. In this single center retrospective cohort study, all lung transplant recipients between November 1990 and September 2020 were analyzed and clinically relevant airway complications of the anastomosis or distal airways were identified and scored according to the ISHLT grading system. We studied potential risk factors for development of airway complications and evaluated the impact on survival. The treatment modalities were described. In 651 patients with 1,191 airway anastomoses, 63 patients developed 76 clinically relevant airway complications of the airway anastomoses or distal airways leading to an incidence of 6.4% of all anastomoses, mainly consisting of airway stenosis (67%). Development of airway complications significantly affects median survival in post lung transplant patients compared to patients without airway complication (101 months versus 136 months, *p* = 0.044). No significant risk factors for development of airway complication could be identified. Previously described risk factors could not be confirmed. Airway stents were required in 55% of the affected patients. Median survival is impaired by airway complications after lung transplantation. In our cohort, no significant risk factors for the development of airway complications could be identified.

## Graphical Abstract

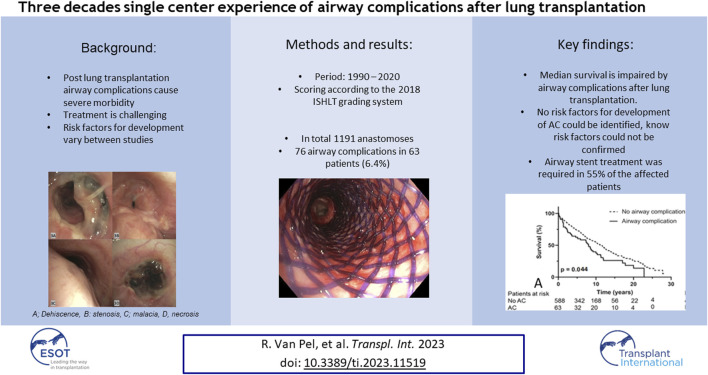

## Introduction

Since the first lung transplantation, anastomotic airway complications (AC) have been a major cause of morbidity and mortality in lung transplant recipients [1, 2]. Broncho-arterial blood supply is not restored during transplantation [3, 4] and viability of the donor bronchus depends on retrograde blood flow. Development of AC can thus be attributed to hypoperfusion of the donor bronchi [5] and is subdivided in stenosis, malacia, dehiscence and necrosis [6]. Multiple grading systems have been developed for scoring AC [6, 7] Data from mainly retrospective cohort studies show AC incidence ranging from 2% to 18% [8–11].

Revascularization of the blood supply can take up to 4 weeks. In this period, ischemia can cascade an inflammatory response with remodeling and risk of both stenosis and malacia [12–15]. To prevent ischemia the donor and recipient bronchus are kept as short as possible [16] to acquire the shortest distance for retrograde bronchial perfusion and have the anastomosis within mediastinal tissue. Risk factors for development of AC are mainly associated with compromised blood flow, such as post-operative hypo perfusion [13] or acute cellular rejection [17]. Risk factors have also been suggested that cannot be related to hypoperfusion directly, for instance right sided anastomosis [9], prolonged ventilation of >48 h of donor [18] and height difference between donor and recipient [11, 18].

Management of AC is diverse and depends on etiology and severity [12]. Endoscopic interventions range from balloon dilatation, electrocautery debridement, laser treatment, cryoablation to endobronchial stent placement [19]. In case of stenosis necessitating treatment, the first approach is (repeated) balloon dilatation which can be sufficient in up to 26% [20]. Recurrent or persistent stenosis, malacia and dehiscence can be treated with endobronchial stent [21] after careful consideration given the potential complications such as sputum statis, infections, stent migration or granulation formation with re-stenosis [19, 22].

In this study, we evaluated known risk factors and try to identify new risk factors for the development of AC. Furthermore, survival data within different treatment modalities of AC were analyzed in our cohort.

## Materials and Methods

### Study Design

Patients with a unilateral or bilateral lung transplantation with an age >18 years transplanted at the University Medical Center Groningen in The Netherlands between 1990 and 2020 were analyzed for AC. Patients gave written informed consent for transplant-related research and this analysis was approved by the local medical ethics committee (METc 2021.00408).

AC was defined as any airway problem necessitating bronchoscopic intervention or follow up. In this study, we graded all AC according to the 2018 ISHLT grading system at the time of detection based on bronchoscopy images and reports as far as possible [6]. With airway malacia defined as >50% reduction in luminal caliber with expiration and clinical impairment. The clinical characteristics of donor and recipient (pre- and post-transplant) were analyzed. Patients who died within 30 days after lung transplantation of causes not related to AC were excluded. From 2005 onward, donation after circulatory death (DCD) donor lungs were accepted besides donation after brain death (DBD) donor lungs.

AC treatment was subdivided into expectative with debridement at most, conservative treatment (balloon dilatation, electrocautery, laser therapy, cryotherapy, mitomycin C application), stent placement or surgical intervention.

The practiced surgical technique has been an end-to-end anastomosis, with telescoping technique in case of anatomical necessity. Since the publication of Aigner et al in 2003 [16], a running suture for the cartilaginous part was introduced. In addition we adopted the practice of a short as possible donor bronchus in 2010 [23]. In case of bilateral transplantation, primary implantation of the right lung is preferred, depending on anatomical variation. According to local protocol, routine bronchoscopy is performed for inspection of the anastomosis during transplantation, prior to extubation and before hospital discharge. Surveillance bronchoscopy was standard at 6, 12, 18, and 24 months until 2008 and is adjusted to bronchoscopy on clinical indication or decline of lung function since. Acute cellular rejection was treated with pulse 1,000 mg methylprednisolone for 3 days. Immune suppression regime evolved over time from rATG, cyclosporine, azathioprine, and prednisolone in the beginning (1990–2001) of the program to Basiliximab, tacrolimus, azathioprine, prednisolone (2001–2009) and Basiliximab, tacrolimus, mycophonolate mofetil and prednisolone since 2009.

Intervention bronchoscopy for stent placement was performed under general anesthesia with rigid or flexible bronchoscope depending on individual case characteristics. Commercially available self-expandable metallic stents (SEMS) were used as standard. From 2019 and on biodegradable stents (ELLA-CS Ltd, Czech Republic) were used.

### Statistical Analysis

Continuous data are expressed as median + range. Nominal variables are expressed as percentages. Because data did not fulfill conditions for normal distribution, non-parametric tests were used. To test for significance in categoric data Pearsons Chi squared test was used or if necessary, Fishers exact test. Continuous data was analyzed with the Mann-Whitney U test. Survival was analyzed with Kaplan-Meier analysis with Log-rank testing. All analyses have been conducted with IBM SPSS Statistics version 23 (IBM, Chicago, USA) and Graphpad Prism 9 (GraphPad software, Inc., La Jolla, USA).

## Results

Between November 1990 and September 2020, 758 lung transplantations were performed. After exclusion criteria 651 patients for our analysis remained. This cohort contained 540 bilateral, 40 unilateral left and 71 unilateral right transplantations resulting in 1191 airway anastomoses. Seventy-six AC occurred in 63 patients, with an AC prevalence of 6.4% per anastomosis and 9.6% per patient. Thirty-eight AC were on the left side and 36 on the right side (*p* = 0.278). The median age for lung transplantation was 52 years for the non-AC population and 50 years for the AC population (*p* = 0.893), 51% of the non-AC population was male compared to 57% of the AC patient group (*p* = 0.264).

**FIGURE 1:**
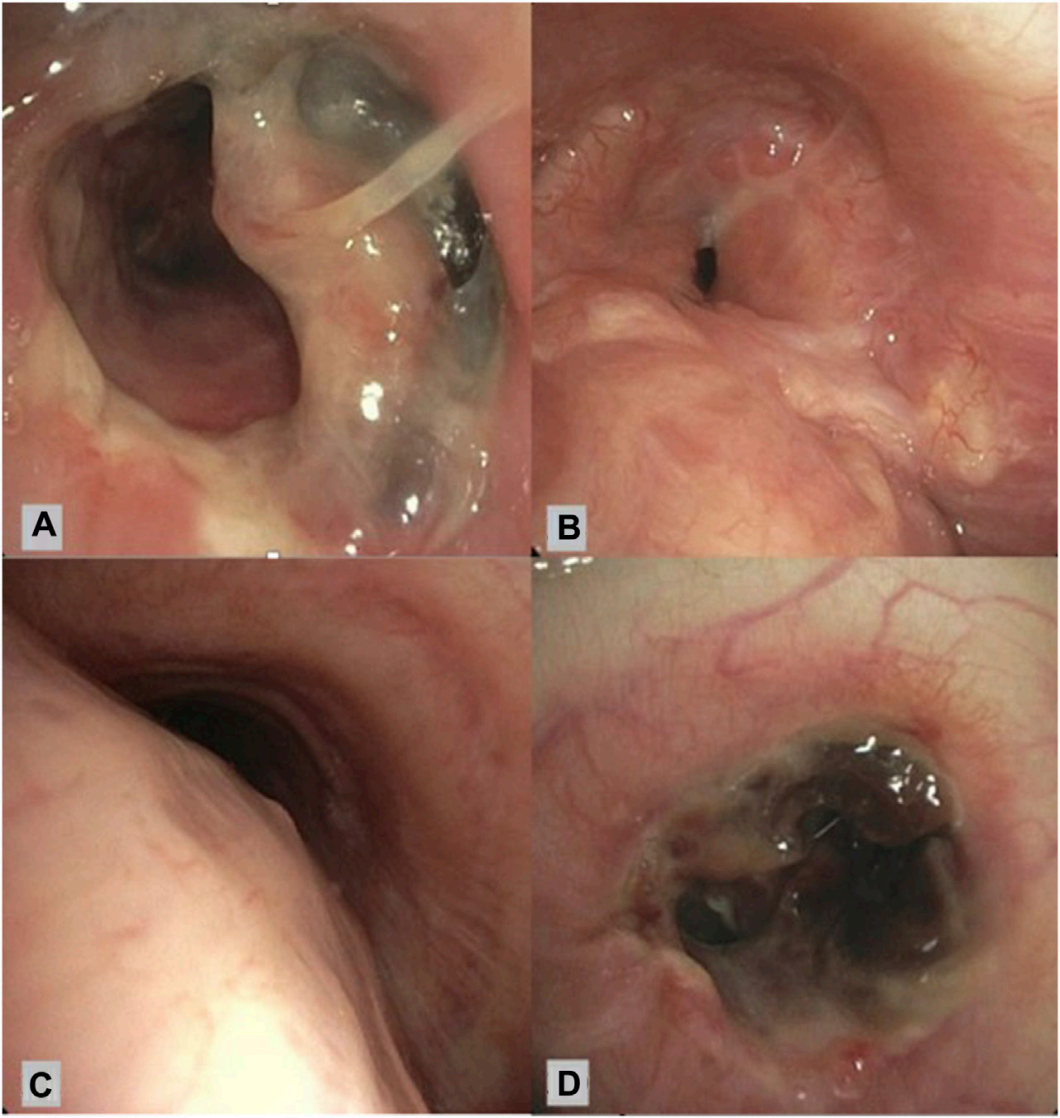
Bronchoscopic view of different airway complications. **(A)**: Partial airway dehiscence at anastomosis ISHLT grading: DLaEb, **(B)**: Airway stenosis at anastomosis ISHLT grading SLaEc, **(C)**: Airway malacia proximal of anastomosis ISHLT grading Mb, **(D)**: Airway necrosis at anastomosis ISHLT grading NLaEd [6].

**FIGURE 2:**
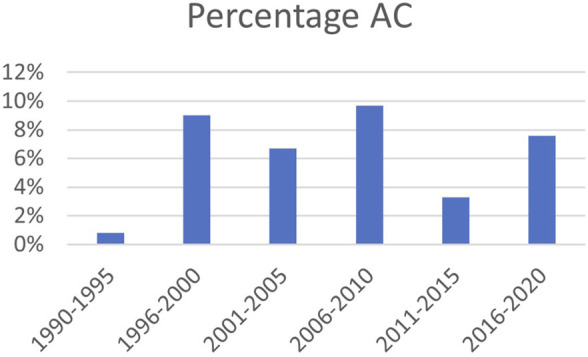
Prevalence of AC over time; percentage of total number of anastomoses. AC: airway complication.

The 76 cases of AC were subdivided into airway stenosis: 51 (67%), malacia: 11 (15%), ischemia/necrosis: 9 (12%) and dehiscence: 5 (7%). Figure 1 shows an example of all four types of AC with corresponding ISHLT grading. Median time until detection of AC was 12 weeks. See Table 1 for the further grading of all AC. 32 of the 51 stenoses consisted of an anastomotic location with >50% but <100% reduction in cross-sectional area. The 11 malacia occurred perianastomotic in 4 cases (36%%) and diffuse in 7 (64%) of the cases. Three of the 5 dehiscence could not be specified besides being partial. Figure 2 shows the prevalence of AC per anastomosis throughout the years.

**TABLE 1:** Distribution according to 2018 ISHLT grading, time until detection and treatment of AC.

Grading			Median time to detection in weeks (range)	Treatment			
				E	C	AS	S
**All (N = 76)**			**12 (1–630)**	**15**	**17**	**41**	**3**
**Ischemia and Necrosis (I) (N = 9)**			5 (1–14)	**0**	**5**	**4**	**0**
Location							
A	Perianastomotic – within 1 cm of anastomosis	1 (11%)	14	0	0	1	0
B	Extending > 1 cm from anastomosis to major airways	6 (67%)	5 (1–8)	0	4	2	0
C	Extending > 1 cm for anastomosis into lobar or segmental airways	2 (22%)	9 (1–14)	0	1	1	0
Extent							
A.	<50% circumferential ischemia	0		0	0	0	0
B.	50%–100% circumferential ischemia	0		0	0	0	0
C.	<50% circumferential necrosis	0		0	0	0	0
D.	>50–100% circumferential necrosis	9 (100%)	5 (1–14)	0	5	4	0
**Dehiscence (D) (N = 5)**			**2 (1–4)**	**4**	**0**	**0**	**1**
Location							
A.	Cartilaginous	2 (40%)	2 (1–3)	1	0	0	1
B.	Membranous	0		0	0	0	0
C.	Both	0		0	0	0	0
	Unknown	3 (60%)	2 (2–4)	3	0	0	0
Extent							
B.	>25–50% circumference	2 (60%)	3 (3–4)	1	0	0	1
	Unknown	3 (60%)	2 (1–2)	3	0	0	0
**Stenosis (S) (N = 51)**			**13 (1–630)**	**6**	**11**	**32**	**2**
Location							
A.	Anastomotic	41 (80%)	10 (1–467)	5	6	28	2
B.	Anastomotic plus lobar/segmental	4 (8%)	328 (4–630)	0	2	2	0
C.	Lobar/segmental only	6 (12%)	13 (2–35)	1	3	2	0
Extent							
A.	0%–25% reduction in cross-sectional area	1 (2%)	5	0	0	1	0
B.	>25%–50% reduction in cross-sectional area	8 (16%)	26 (2–467)	3	3	2	0
C.	>50%–100% reduction in cross-sectional area	40 (78%)	11 (2–630)	3	8	28	1
D.	100% obstruction	2 (4%)	4 (1–7)	0	0	1	1
**Malacia (M) (N = 11)**			**36 (9–340)**	**5**	**1**	**5**	**0**
A.	Perianastomotic – within 1 cm of anostomosis	4 (36%)	36 (25–280)	1	0	3	0
B.	Diffuse – involving anastomosis and extending beyond 1 cm	7 (64%)	44 (9–340)	4	1	2	0

E, Expectative treatment include debridement at most; C, conservative treatment consisting of balloon dilatation, incision, laser therapy, cryotherapy and mitomycin application; AS, airway stent; S, surgery.

### Risk Factors for Development of Airway Complications

#### Donor Characteristics

Median donor age was 45 years for non-AC patients versus 46 for AC patients (*p* = 0.896). Median donor age increased from 36 (range 12–55) years in the first 5 years of the program to 51 (range 11–78) in the most recent 5 years. Median ventilation time was 2 days for both AC affected, and non-AC affected patients (*p* = 0.872). Median donor packyears was 0 years for AC patients and non-AC patients alike (*p* = 0.693) (Table 2). Donation after circulatory death (DCD) was introduced in 2005 and performed in 32% of the transplants since and totals 21% of the entire cohort. AC occurred in 13 out of 126 (10%) of the DCD patients and 50 out of 462 (11%) of the DBD patients (*p* = 0.884). Donor ventilation time >48 h was not a risk factor for development of AC (*p* = 0.992). Median donor age was 30 years in the period from 1990 to 1995 and 51 in the period from 2015 to 2020. Forty patients were pretreated with *ex vivo* lung preservation, incidence of AC in this group was comparable to the cases without this treatment (*p* = 0.714).

**TABLE 2:** Lung transplant donor related characteristics.

	All N = 651	AC N = 63	No AC N = 588	Sign
Donor age	46 11–78)	46 (18–71)	45 (11–78)	*p* = 0.896
Donor ventilation in days	2 (0–41)	2 (0–11)	2 (0–41)	*p* = 0.872
Donor height in centimeters	175 (120–196)	176 (158–190)	174 (120–196)	*p* = 0.281
Donor packyears	0 (0–50)	0 (0–25)	0 (0–50)	*p* = 0.693
Donation after circulatory death	139 (21%)	13 (21%)	126 (21%)	*p* = 0.884

AC, Airway complication. Continuous variables are expressed as median (range).

#### Recipient Characteristics

The most common indication for lung transplantation was chronic obstructive pulmonary disease *n* = 301 (42%) followed by interstitial lung disease *n* = 132 (20%), infectious pulmonary disease including cystic fibrosis and bronchiectasis *n* = 116 (18%), and pulmonary vascular disease *n* = 52 (8%). Median donor-recipient size mismatch was −1 cm for both the AC patients and the non-AC patients (*p* = 0.980, ns). After introduction of the running suture for the membranous part in 2003, occurrence of AC was stable (7.4% vs. 12%, *p* = 0.129). Median recipient age raised from 45 years (range 19–64) in the first 5 years of the program to 58 (range 19–68).

Median intensive care unit (ICU) admission was 6 days for both AC patients and non-AC patients (*p* = 0.209). Median time to extubation was 2 days for both AC patients and non-AC patients (*p* = 0.095). Sixty seven of the 76 (88%) AC were identified after ICU discharge.

Surgical Ischemic time for implantation of the first lung was 313 min for patients with AC and 314 min for patients without AC (*p* = 0.814). Lung transplants were performed by twenty five surgeons, with a median of 28 transplants per surgeon (range 1–98). No difference in incidence of AC was observed between high volume (N > 28) and low volume (N < 28) surgeons (*p* = 0.515).

238 (40%) of the non-AC patients were treated for acute rejection within 30 days post-transplant compared to 31 (49%) of the AC patients (*p* = 0.115). Development of primary graft dysfunction of any severity whatsoever occurred in 313 (53%) of the non-AC patients and 34 (54%) of the AC patients (*p* = 0.734). The risk for development of AC was not influenced by the immune suppression regime (*p* = 0.162). See Table 3 for further recipient characteristics.

**TABLE 3:** Lung transplant recipient related characteristics.

Recipient characteristics	All = 651	AC N = 63	No AC N = 588	Sign
Age lung transplant	52 (19–69)	50 (19–66)	52 (19–69)	*p* = 0.893
Male	328 (51%)	36 (57%)	292 (50%)	*p* = 0.264
Underlying disease:				
Obstructive pulmonary disease	301 (42%)	29 (43%)	282 (42%%)	*p* = 0.410
Infectious pulmonary disease	116 (18%)	10 (16%)	106 (18%)	
Vascular pulmonary disease	52 (8%)	2 (3%)	50 (9%)	
Insterstitial pulmonary disease	132 (20%)	16 (26%)	116 (18%)	
Other	44 (7%)	6 (10%)	38 (6%)	
Length (centimeter)	172 (149–198)	174 (160–194)	172 (149–198)	*p* = 0.914
Treatment for acute rejection first 30 days post-transplant	269 (41%)	31 (49%)	238 (40%)	*p* = 0.115
Any primary graft dysfunction in first 72 h	347 (53%)	34 (54%)	313 (53%)	*p* = 0.734
Time of ischemia of first implanted lung	314 (93–1,137)	313 (93–861)	314 (105–1,137)	*p* = 0.814
Time of ischemia second implanted lung (if applicable)	444 (158–1,271)	457 (158–989)	443 (197–1,271)	*p* = 0.877
ICU length of stay	6 (0–158)	6 (1–126)	6 (0–158)	*p* = 0.209
Time to extubation in days	2 (0–158)	2 (0–52)	2 (0–158)	*p* = 0.095
Size mismatch donor-recipient cm	−1 (-29–35)	−1 (-13–16)	1 (-29–35)	*p* = 0.980

AC, airway complication; ICU, intensive care unit. Continuous variables are expressed as median (range).

Treatment; Table 1 shows the treatment strategy of all 76 AC. Fifteen (20%) were approached expectative with follow up bronchoscopy or debridement at most without further intervention. AC was treated with conservative therapy in 17 (22%) of the cases.

Treatment with airway stent was required in 42 (55%) of the AC in 36 patients. Twenty-six airway (65%) stents were placed in the left main bronchus, fourteen (35%) in the right main bronchus and bronchus intermedius. Six cases were treated with biodegradable stent, 5 after prior treatment with SEMS, which were removed before biodegradable stent placement.

Three surgical interventions were required. One for a partial dehiscence, one pneumonectomy for stent therapy refractory stenosis and one anastomosis revision for stenosis without prior treatment with airway stent.

**FIGURE 3:**
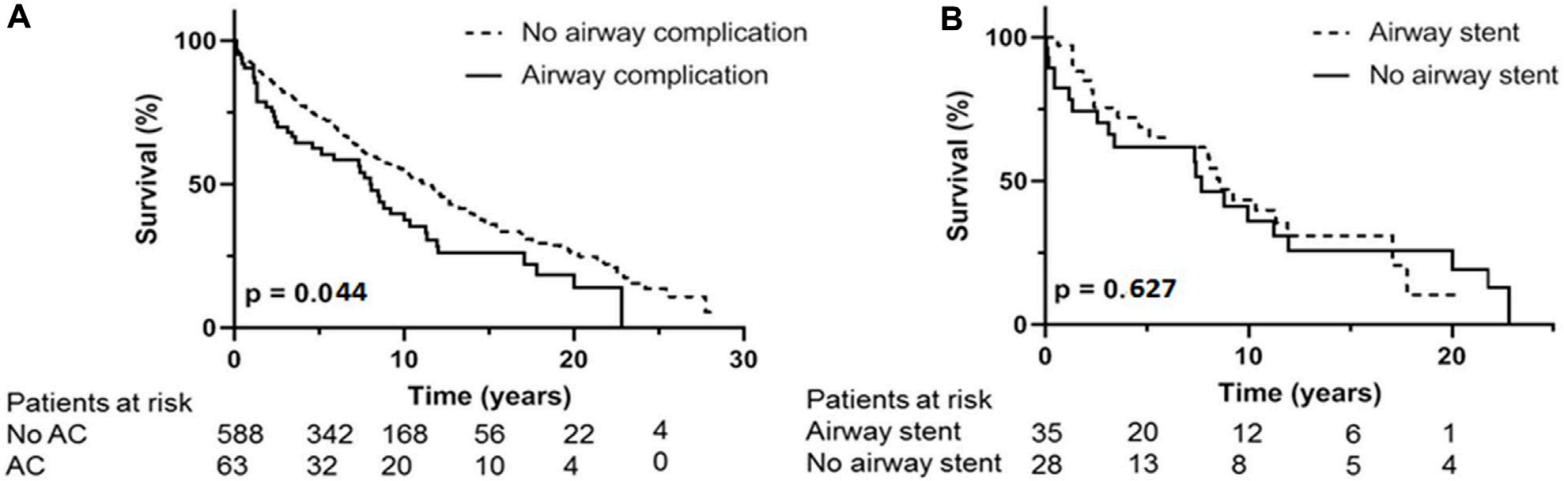
**(A)**: Kaplan Meijer estimates for survival. **(A)** occurrence of airway complication compared to no airway complication. **(B)** analysis within AC group, difference in survival between treatment with airway stent compared to treatment without with airway stent. AC: airway complication.

Survival; Figure 3A shows the overall survival between the 63 AC and the 588 non-AC lung transplant recipients. Occurrence of AC led to significantly shorter survival with median survival of 101 months versus 136 months (*p* = 0.044). Forty one of the 63 patients with AC died in the follow up. Cause of death could not be related to AC or its complications in 35 (85%) of the patients including 7 patients dying of chronic allograft dysfunction and 8 patients dying of a malignancy. One patient died because of a dehiscence of the right main bronchus without further treatment options, one patient with airway dehiscence died due to multi organ failure. Five patients with SEMS *in situ* died of pulmonary infection with *Aspergillus Fumigatus* and/or *Pseudomonas aeruginosa.*

In the 5 patients with airway dehiscence the median survival was 1 month (range 0–88 months). When these cases are excluded from survival analyses, median survival was not significantly different, but still showed a trend in favor of the non-AC group, with a median survival of 105 months for AC patients and 136 months for non-AC patients (*p* = 0.142).

Within the patient group suffering from AC, necessity of endobronchial airway stent placement shows overall median survival of 102 months compared to median survival of 91 months in AC patients without stent placement (*p* = 0.627) (Figure 3B). The 36 patients receiving an airway stent had a median survival of 102 months compared to 132 months in the remaining 615 patients in the total cohort (*p* = 0.346).

## Discussion

In our retrospective cohort study investigating our entire 30 years’ experience of lung transplantation, the incidence of clinically relevant AC post lung-transplant was 6.4% per anastomosis. No significant risk factors were identified of development of AC. However, occurrence of AC was associated with worse survival.

Incidence of AC was comparable to similar studies [8, 14, 17, 18] and surprisingly the incidence of AC did not decrease over time. Despite advances in surgical techniques, organ preservation and decrease in rejection. A recent study from the Vienna lung transplant center [24], which has a very high transplant volume, did show a low incidence of 1.56%. This indicates that experience might be beneficial, though the incidence of AC in our cohort was similar between experienced and less experienced surgeons.

Another explanation can be the increased acceptance of recipients with more comorbidities and higher age. In our series, the median donor age increased from 30 years in the first 5 years of the program to 51 years in the most recent 5 years.

The AC have been scored according to the most recent ISHLT grading system [6]. What argues for the use of this grading is the rapid detection of necrosis. Necrosis often predisposes stenosis and malacia [6], and early recognition and debridement might prevent development of stenosis and malacia. However, structural recognition of early onset of necrosis asks for structural and periodic endobronchial inspection. This is not standard care in our- and most-institutions, which raises the question how feasible the classification systems are in day-to-day clinical setting. Given the fact that median detection of AC occurred at 12 weeks post-transplant and most AC have already been developed to a significant stenosis or malacia at the time of detection this is often after the period of standard periodic inspection. This is emphasized by the fact that all clinically significant cases of necrosis/ischemia had a 50%–100% circumferential necrosis. It is plausible that necrosis and ischemia occur much more frequently, but that this does not cause clinical complaints and may only become apparent after organization to stenosis.

### Risk Factors Associated With Airway Complications

There are conflicting data on the relation between acute rejection and development of AC [11, 13, 17, 25]. In the cyclosporine era (1990–2001) a high number of patients were treated for acute rejection leading to 41% off all lung transplant patients treated in the first 30 days post transplantation. This is high compared to other studies [13, 17]. But did not result in more AC (*p* = 0.115).

The median ischemic time in this study is 314 min for the first implanted lung and 444 min for the second if applicable. We did not find a relation between ischemia time and development of AC in contrast to recent studies. However, these are studies with average longer ischemia time compared to our cohort, ranging from 354 min for single and 516 for double lung transplantation [26, 27]. This study does not confirm previous reports that right sided anastomosis [9] is a risk factor for development of AC, previously attributed to bronchial artery anatomy. A possible explanation could be that in our center right lung implantation is preferably done first leading to a shorter time of ischemia, although this is common practice in most lung transplantation centers.

Findings in this study argue against previous studies that prolonged mechanical donor ventilation time is associated with higher incidence of AC [18]. Height mismatch between donor and recipient neither showed to be a risk factor for development of AC.

From the start of the transplant program, surgical technique has been the same with the end-to-end technique with separate single sutures for the membranous part with introduction of a running suture for the cartilaginous part after 2003. Therefore, no comparison can be made between surgical techniques. Heart-lung transplantations have been excluded because of the tracheal anastomosis and the possible decreased risk of development of AC attributed to collateral vessels from the coronary arteries [5].

In our institution we have a high percentage (31%) of DCD lungs since introduction in 2005 compared to other comparable studies [9]. Yet, this did not lead to a higher risk for development of AC. conform the aforementioned study [9].

Airway stent placement was required in 54% of the patients compared to 12%–44% in comparable studies [8, 18]. The high incidence of endobronchial stent placement strengthens the hypothesis of a more severe affected patient population. Traditionally, mainly SEMS are used [19] with silicone stents as alternative [28]. Endobronchial stent placement is associated with complications as sputum stasis, stent migration, in stent stenosis and infectious complications [29]. Recently, bio-degradable stents have been developed and small case series have proven feasibility [30, 31]. Considering that the need for endobronchial stent is often temporary and stent removal is associated with possible complications [19, 28] the use of bio-degradable stents could be a less invasive alternative.

This study showed significant impact on survival for patients affected with AC. With median survival significantly reduced from 136 to 101 months. This in contrast with data from previous studies [8, 14, 17] which show no influence on survival. Dehiscence seems to play an important role in this finding because when these cases are excluded median survival only shows a non-significant trend to worse survival (105 vs. 136 months, *p* = 0.142). It is known that dehiscence is associated with worse outcome [6, 24], though in previous literature these cases are systematically counted to the AC and are included in survival analyses.

Within the group of patients treated with a SEMS. Five patients died secondary to pulmonary infection with *Staphylococcus Aureus* and/or *Aspergillus fumigatus.* In 2009, Gottlieb et al. [22]*,* already showed a strong association between SEMS and bacterial colonization and, in their analysis, there was a significant effect on survival. In this cohort, treatment with airway stent did not show impaired survival when compared to the AC group (*p* = 0.489) or the overall post lung-transplant cohort (*p* = 0.200). In the clinical practice, particularly these AC will be identified when symptoms as dyspnea and loss of lung function occur, and it is impossible to predict which patient will be affected without clear risk factors. Therefore, new developments of endobronchial treatment of AC are even more important, for instance with biodegradable airway stents to both avoid granulation and infection issues.

Limitations of this study are the retrospective character. Furthermore, the endobronchial treatment techniques have developed over the years with more treatment options. Nonetheless, this study provides a good reflection of the clinical reality and challenges associated with AC.

## Conclusion

In our study we could not confirm any of the previous described risk factors for development of AC and found no new risk factors. Assuming risk factors for development of airway complications are unclear, and therefore virtually impossible to influence. Future research should focus on improving treatment of AC, for example with pro-active bronchoscopic maintenance of the anastomosis region to avoid stent necessity and if biodegradable endobronchial stents prevent colonization at the stent site in comparison to SEMS.

## Data Availability

Requests to access the datasets analyzed in this study should be directed to r.pel@erasmusmc.nl.
